# Fruit and Vegetable Intake Assessed by Repeat 24 h Recalls, but Not by A Dietary Screener, Is Associated with Skin Carotenoid Measurements in Children

**DOI:** 10.3390/nu13030980

**Published:** 2021-03-18

**Authors:** Rebecca A. Seguin-Fowler, Karla L. Hanson, Grace A. Marshall, Emily H. Belarmino, Stephanie B. Jilcott Pitts, Jane Kolodinsky, Marilyn Sitaker, Alice Ammerman

**Affiliations:** 1 Texas A&M AgriLife Research and the Department of Nutrition, Texas A&M University System, College Station, TX 77843, USA; 2 Master of Public Health Program and the Department of Population Medicine and Diagnostic Sciences, Cornell University, Ithaca, NY 14853, USA; kh289@cornell.edu (K.L.H.); gam263@cornell.edu (G.A.M.); 3 Department of Nutrition and Food Science, University of Vermont, Burlington, VT 05405, USA; Emily.Belarmino@uvm.edu; 4 Department of Public Health, East Carolina University, Greenville, NC 27834, USA; jilcotts@ecu.edu; 5 Community Development and Applied Economics Department, University of Vermont, Burlington, VT 05405, USA; Jane.Kolodinsky@uvm.edu; 6 The Evergreen State College, Olympia, WA 98505, USA; msitaker@gmail.com; 7 Department of Nutrition, University of North Carolina at Chapel Hill, Chapel Hill, NC 27599, USA; alice_ammerman@unc.edu

**Keywords:** fruit and vegetable consumption, skin carotenoids, 24 h recalls, fruit and vegetable screener, children, low-income US households, resonance Raman spectroscopy

## Abstract

Accurate measurement of fruit and vegetable (FV) intake is important for nutrition surveillance and evaluation of dietary interventions. We compared two tools for reporting FV intake to objective measurement of skin carotenoids among children. FV cups/day was assessed by repeated 24 h dietary recalls (24H FV) and the National Cancer Institute’s All-Day Fruit and Vegetable Screener (NCI FV). Skin carotenoids were measured by repeated resonance Raman spectroscopy (RRS) of the palm. FV cups were regressed on RRS scores in unadjusted, field-based, and research-setting models with covariates feasible in each scenario. Data were baseline values from children aged 2–12 years in low-income households enrolled in a healthy eating randomized trial in four U.S. states (*n* = 177). Twenty-four-hour FV cups were associated with skin carotenoids in all models (*p* < 0.001) but NCI FV cups were not. Predicted RRS scores for discrete 24H FV cups provide a guide to interpretation of RRS in children (2 cups FV intake ~36,000 RRS units), with the research-setting scenario generally providing the narrowest prediction range (+/−1924). When self-reported data are required, 24 h recalls are more accurate than NCI FV screener data; and, when limited time, resources, or literacy must be considered, RRS scores can be quickly obtained and easily interpreted.

## 1. Introduction

A diet rich in fruits and vegetables (FV) is associated with reduced risk for many diseases [1,2]. However, most children in the U.S. do not meet recommendations for FV intake [3]. In fact, from 2003 to 2010, no socio-demographic sub-group of children met the U.S. Healthy People 2020 total vegetable target, and only children aged 2–5 years met the fruit target [3]. Because dietary patterns established in childhood persist into adulthood [4], a large number of policies and programs aim to encourage children to eat more FV. Accurate assessment of children’s intake is critical to judge progress toward this goal.

Measurement of children’s diets is challenging because most methods depend on participants’ reports; typically, older children self-report and caregivers report on behalf of younger children [5]. Errors ensue due to children’s cognitive abilities, motivation, and reporting biases, as well as reliance on caregivers’ knowledge of what young children eat [6]. Further, while daily methods such as 24 h recalls and food records are comprehensive, the time and resources required to complete recalls may limit their suitability for community-based surveillance and intervention evaluation [7]. Dietary screeners are quick but provide less information. Thus, there is demand for valid, rapid, and less burdensome methods to assess children’s dietary intake in a variety of settings.

Carotenoids are naturally occurring, fat-soluble organic compounds responsible for pigmentations found in FV [8]. Because FV are a primary source of carotenoids in the diet, carotenoid concentrations in blood and tissues serve as biomarkers for FV intake [9]. Foods rich in carotenoids include fruits such as oranges, cantaloupe, and tomatoes and vegetables such as broccoli, carrots, spinach, and other dark leafy greens [9]. The recent development of sensitive optical technologies has made noninvasive measurement of skin carotenoids possible. One such technology, resonance Raman spectroscopy (RRS), uses a laser at blue wavelength to excite tissue carotenoids and measure the light scattered from the skin to produce an estimate of total carotenoids [10,11]. RRS measurements can be taken with a portable device in about 30 s and provide immediate results. A recent systematic review of studies examining the validity of skin carotenoid measurement included six studies of RRS with children [12,13,14,15,16,17] and reported consistent correlation of RRS scores with total serum or plasma carotenoids [18].

Many factors contribute to skin carotenoid concentrations among adults, including diet, adiposity, genetics, smoking, oxidative stress, sun exposure, general health, and possibly skin pigmentation [10,19,20]. Less is known about which factors contribute to skin carotenoid measurements in children [13,17,21,22], but limited evidence suggests that age, sex, race, and adiposity are relevant. Age, race, cholesterol levels, energy and fat intake, and body mass index (BMI) are commonly included in models examining the association between FV intake and skin carotenoids [14,16,17,21,23].

The present study examined associations between skin carotenoids measured by RRS and child FV intake reported using two different validated tools under different adjustment models. The tools used to assess FV intake in this study were the National Cancer Institute’s (NCI) All-Day Fruit and Vegetable Screener, which queries FV intake over the past month, and three 24 h recalls, which query FV intake on multiple days during a two-week period. Our aim was to assess the strength and significance of associations between each tool and objectively-measured skin carotenoids and to make recommendations about the use of each measurement tool in various settings. Further, because interpretation of RRS scores can be difficult, we provided estimates for interpretation of RRS scores with respect to FV intake among children.

## 2. Materials and Methods

This cross-sectional analysis used baseline data for children aged 2–12 years who participated in a family-based healthy eating intervention in four U.S. states (New York (NY), North Carolina (NC), Vermont (VT), and Washington (WA)) [24]. Households were eligible if they met a low-income threshold (≤185% of the U.S. federal poverty level), included a child 2 to 12 years old and a caregiver who spoke and read English and had access to a computer. A total of 685 households were assessed for eligibility, of which 143 were ineligible (20.9%) and 237 declined to participate (34.6%). The remaining 305 households enrolled. Data for 177 children (58.0%) were retained for this analysis. Children were excluded if they did not have 2 or 3 valid 24 h recalls (*n* = 59) or were missing skin scan (*n* = 36), FV screener (*n* = 3), race (*n* = 27), or ethnicity (*n* = 3). The study protocol was approved by Institutional Review Boards at Cornell University and the University of Vermont (ID numbers: 1501005266; CHRBSS 16-393). All caregivers provided written informed consent and children aged 6–12 years provided oral assent.

Data were obtained through online questionnaires, 24 h dietary recalls, and in-person physical measurements between March and June of 2016 and 2017. All data were reported by the caregiver, with 61% of children ages 6–12 years old assisting with dietary reporting. Caregivers reported the child’s age, sex, race, and ethnicity. Six race options were collapsed into white (reference category), Black/African American, and multi-racial/other. Caregivers also rated the child’s general health status from poor to excellent and reported smokers in the household.

Two tools were used to measure FV consumption, with white potatoes subsequently omitted because they contain few carotenoids [25]. First, the National Cancer Institute’s (NCI) All-Day Fruit and Vegetable Screener asked about intake frequency and portion sizes for nine categories of FV eaten over the past month. The paper-based instrument was converted into an online questionnaire with visual aids added to improve portion estimation [26]. A scoring algorithm was applied to estimate daily cup equivalents of FV intake (NCI FV cups), including imputation of missing item responses [27]. The NCI screener has been validated for use with adults [28,29,30].

Second, caregivers were asked to complete three 24 h recalls for the child on two weekdays and one weekend day during a two-week period using the online automated self-administered 24 h dietary assessment tool [31]. Dietary recalls were considered outliers and were excluded if kilocalories <100 or ≥300% of estimated energy requirement (EER) given age and sex and assuming a moderate activity level [32] or if they included alcohol. Mean reported FV in cup equivalents (24H FV cups) was calculated. Total energy was calculated as mean total kilocalories as a percentage of EER. Fat intake was calculated as the percentage of energy from fat.

Height and weight (without shoes) were measured in duplicate or triplicate if the difference between the first two measurements was greater than 0.125 inches or 0.4 pounds. Mean height and weight were used to calculate body mass index (BMI) as a percentile relative to growth charts based on sex and age (BMI-for-age percentile) [33]. Skin carotenoids were measured using RRS of the palm. Each child was scanned twice using the Pharmanex S3 BioPhotonic Scanner (NuSkin, Provo, UT, USA) or three times if readings differed by more than 2000 units. The mean of all RRS measurements were used in analyses.

Sample characteristics are presented in Table 1 and are summarized in pooled data and by state. FV intake and skin scan scores were examined for normality (skewness and kurtosis within ±2), and measures of FV intake were logged to meet these standards. Multiple linear regression was used to assess associations between logged measures of FV intake and mean RRS scores. Three regression models examined performance when (1) unadjusted, (2) adjusted for covariates available in a field-based scenario (age, race, ethnicity, state, and a smoker in the household), and (3) with additional controls available in research-setting scenario (added BMI-for-age percentile, energy intake, and fat intake). Regression coefficients and *t*-tests for all independent variables were reported. *F*-tests of the change in *R*^2^ across models tested which model best predicted skin carotenoids. Skin carotenoid values associated with discreet 24H FV cup values were estimated using the three regression models, and margins of error were constructed around each prediction. Analyses were conducted using SPSS Statistics version 25 (IBM Corp., Armonk, NY, USA) and SAS version 9.4 (SAS Institute, Inc., Cary, NC, USA).

## 3. Results

On average, children were 5.9 years, most were non-Hispanic white (74.0%), in good or better health (97.2%), and lived in a non-smoking household (84.2%; Table 1). On average, children had slightly higher than average BMI (66.8th percentile) and consumed more energy than needed to meet metabolic requirements (114.6% of EER). On average, children in the sample consumed 2.7 to 3.7 cups of FV/day, depending on the assessment tool used. Race and Hispanic ethnicity differed significantly between states: NC had significantly more Black/African American (40.9%, *p* < 0.001) and Hispanic children (18.2%, *p* < 0.01) than the other states (which included almost none; data not shown). No 24 h recalls reported intake of carotenoid-containing supplements.

In unadjusted models, NCI FV cups were not associated with skin carotenoids, whereas 24H FV cups were positively associated with skin carotenoids (*p* < 0.01, Table 2). Similar results were observed in adjusted models. The models adjusting only for field-based covariates explained 16–20% of variation in skin carotenoids and consistently outperformed the unadjusted models (*p* < 0.001). Age was inversely associated with skin carotenoids (*p* < 0.01), whereas sex, race, ethnicity, and presence of household smokers were not associated with skin carotenoids. Residence in NC, compared to residence in NY or VT, was inversely associated with skin carotenoids (*p* < 0.05).

When research-setting covariates were added to regression models, slightly more variation in skin carotenoids was explained (19–25%) which was a significant improvement when examining 24H FV cups (*p* < 0.05). Dietary fat intake was positively associated with skin carotenoids (*p* < 0.05), whereas BMI and energy intake were not associated with skin carotenoids.

**Figure 1:**
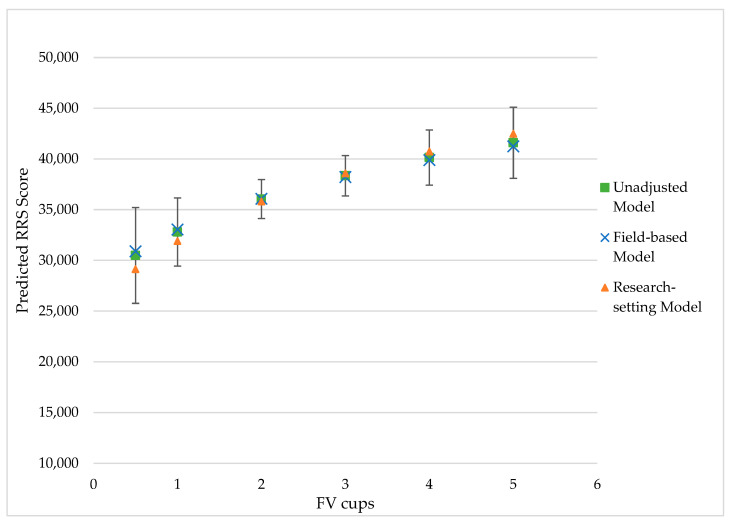
Resonance Raman spectroscopy (RRS) scores predicted by reported FV consumption among children under three scenarios (*n* = 177). FV cups were modeled from 24 h recall data. The unadjusted model included no covariates. The field-based model included adjustments for age, race, ethnicity, state, and a smoker in the household. The research-setting model included all covariates in the field-based model with additional adjustment for BMI-for-age percentile, energy intake, and fat intake. Error bars presented are from the research-setting model.

Predicted skin carotenoid values increased as 24H FV cups increased in all models, and predictions across models were quite consistent (Figure 1). For example, the predicted skin carotenoid value for two 24H FV cups was approximately 36,000: ranged from 36,072.2 unadjusted to 35,814.1 in the research-setting model (Table 3). The margin of error around RRS predictions for two FV cups decreased slightly as more covariates were added to the model (ranged from ±2066.4 unadjusted to ±1924.8 in the research-setting model). At four 24H FV cups, predicted RRS score was approximately 40,000 across models, but the predictions and margins of error did not consistently improve as more covariates were added to the models.

## 4. Discussion

This study found significant positive associations between children’s FV intake measured by 24 h recalls and skin carotenoids measured by RRS in a sample of children from low-income households for whom mean FV intake was relatively high. To the best of our knowledge, only two previous studies compared FV intake measured by 24 h recall and skin carotenoids in children, and both found moderate associations [15,16]. This study uses the largest age-diverse sample of children from multiple, geographically diverse U.S. states to date. These results are in agreement with a recently published meta-analysis that found significant correlations between plasma and skin carotenoids in both children and adults [34]. Taken together, results from our study and prior studies suggest that skin carotenoid assessment is a valid, reliable indicator of FV intake in an age-diverse sample of U.S. children.

Because carotenoids linger in the skin [35,36], the association of skin carotenoids with usual dietary intake assessed by the NCI FV screener was expected to be stronger than the association with short-term FV intake captured by 24 h recalls. However, no significant association was observed between NCI FV cups and skin carotenoids in any models. This is consistent with two other studies that found no significant associations between NCI FV cups and blood [37] or skin [38] carotenoids among adults. Together these results suggest that the NCI FV screener is not a valid measure of FV intake.

In part this may be due to limits on human memory, as caregivers may have difficulty recalling what their child ate over the last month, which may introduce measurement error. Differences have also been observed between adults’ FV intake measured by the FV screener and 24 h recalls [39]. This study used best practices in adapting and implementing the FV screener in this study (easy on-line completion, photos of portion sizes, high child participation in dietary reporting) to address measurement limitations [26]. Still, only FV intake data measured using the 24 h recall tool was significantly associated with objective measurement of skin carotenoids.

Multivariate regression modeling revealed significant associations between age, state of residence, and fat intake with skin carotenoids. Age was inversely associated with skin carotenoids, in contrast to one prior study that suggested a direct relationship between age and skin carotenoids in a sample with a narrow range of ages (3–5 years old) [22]. Previous research identified inverse associations between age and carotenoid concentrations in adults, but these associations are not well understood [20]. Evidence suggests that younger children consume more FV than older children [40,41], but it is also unknown whether children absorb or metabolize carotenoids differently than adults [13] or differently at unique stages of development. Any of these factors may have contributed to the observed associations between age and skin carotenoids.

There were inverse associations between skin carotenoids and residence in NC relative to NY in all adjusted regression models. These associations may reflect state differences in intake of foods containing carotenoids, but sun exposure and racial/ethnic background of participants across states may also may have contributed to these associations. Sun exposure has been associated with lower skin carotenoids in adults [35,36]. Although skin carotenoid measurements were timed to eliminate seasonal variation in sun exposure, NC is further south and has more clear days from March to June compared to NY, VT, and WA, which are relatively similar in latitude and sunshine [42,43]. Three prior studies explored the question of whether skin pigmentation is associated with lower skin carotenoid measurement, but limitations in sample size and diversity precluded a definitive answer [19,38,44]. Our sample was also limited in racial/ethnic diversity, with NC having the highest proportions of Black/African American and Hispanic children compared to other states. Most prior studies of skin carotenoids in children used homogeneous samples [12,13,15,16], with only one study reporting more diversity in a large sample [17]. Small sample sizes limited our ability to fully explore the roles of sun exposure and skin pigmentation on measurement of skin carotenoids in this study.

For each measure of FV intake, controlling for socio-demographic characteristics explained significantly more variation in skin carotenoids than before adjustment. However, additional controls created only slight improvement in explanatory power. This suggests that RRS is appropriate for use in public health surveillance and evaluation with or without additional controls, such as BMI or intakes of energy and fat.

As expected, higher skin carotenoid values were predicted for a greater number of FV cups estimated by two or three 24 h recalls. These predicted RRS scores for discrete FV cups provide a guide to interpretation of RRS in children (e.g., 2 cups FV intake ~36,000 RRS units). Furthermore, little variation was observed between the predicted RRS scores from each of the three models, suggesting that this “crosswalk” could be used to estimate FV cups from skin carotenoid values in a variety of research situations. The margins of error were generally larger using the unadjusted model, suggesting that the use of available or readily collected covariates should be considered in all settings.

Skin carotenoid levels measured by RRS have been validated in adults and children [18,34], making skin carotenoids an important objective biomarker for FV intake. Furthermore, studies with adults suggest that skin carotenoids measured by RRS are more strongly associated with serum carotenoids than they are with any participant-reported measures of FV [18]. Measurement of skin carotenoids as a biomarker for FV intake may be appropriate for a broad range of community and clinical settings, including those in which time, resources, or literacy are limited. We suggest that future public health nutrition programs consider measuring skin carotenoids as a valid indicator of FV intake and changes when it is feasible to do so. We recommend measuring skin carotenoid levels with RRS as a quick assessment approach that can be easily interpreted using the RRS ranges associated with discrete cups of FV from our models. Further, this study suggests that when objective measurement of skin carotenoids in children is infeasible, FV intake calculated from two or three 24 h recalls in children is a viable alternative, but FV intake estimated from the NCI FV screener is not.

Future research should refine estimates of skin carotenoids (using tools such as RRS) for discrete FV cup measurements by including larger samples. Research should also explore the roles of sun exposure and skin pigmentation on skin carotenoid measurement by including sites geographically diverse in latitude and climate, with racially and ethnically diverse samples. Continued testing of brief, noninvasive tools for objective measurement of biomarkers for FV intake (such as RRS) among children would help advance our understanding of their utility for nutrition surveillance and evaluation of interventions to improve dietary quality among children.

## Figures and Tables

**Table 1:** Characteristics of children enrolled in a healthy eating intervention trial in four U.S. states (*n* = 177).

	Mean	SD	Count	Percent
**Characteristics**				
Age (years)	5.9	2.8		
Sex				
Female			94	53.1
Male			83	46.9
Race				
Black/African-American			15	8.5
White			131	74.0
Multi-racial/other ^1^			31	17.5
Hispanic			9	5.1
State				
New York			66	37.3
North Carolina			22	12.4
Vermont			50	28.2
Washington			39	22.0
**Health-related Factors**				
General health status				
Excellent			74	41.8
Very good			68	38.4
Good			30	16.9
Fair			5	2.8
Poor			0	0.0
Smoker(s) in household			28	15.8
BMI-for-age percentile	66.8	30.2		
**Dietary Intake**				
Total FV intake (NCI FV cups)	3.7	3.5		
Total FV intake (24H FV cups)	2.7	1.5		
Total energy intake (%EER) ^2^	114.6	33.5		
Daily fat intake (%kcal)	34.4	6.4		

BMI, body mass index; FV, fruit and vegetable; NCI FV cups, National Cancer Institute’s All-Day Fruit and Vegetable Screener cup equivalents; 24 H FV cups, mean 24 h recall fruit and vegetable cup equivalents; EER, estimated energy requirement; kcal, kilocalories; SD, standard deviation. ^1^ Other includes 5 American Indian/Alaskan Natives, 1 Asian/Pacific Islander, and 4 people who reported their child was none of the options offered. ^2^ Calculated assuming a moderate level of physical activity.

**Table 2:** Associations between two measures of FV intake and skin carotenoids among children (*n* = 177).

	Unadjusted Model		Field-Based Model		Research-Setting Model	
	β	SE	β	SE	β	SE
Log NCI FV cups	2265.7	1737.3	1067.4	1673.9	1198.7	1679.4
Age (years)			−1111.2 **	340.3	−1042.6 **	341.7
White (ref)						
Black/African-American			−1435.0	4000.0	−1741.6	3991.0
Multi-racial/other			1341.7	2581.4	796.3	2575.3
Hispanic			−3360.0	4675.7	−2725.3	4660.5
New York (ref)						
North Carolina			−11,660.8 **	3520.3	−11,944.6 ***	3529.6
Vermont			−2295.6	2437.0	−2817.0	2427.5
Washington			−5710.1 *	2593.8	−6236.6 *	2616.5
Smoker(s) in household			−930.7	2659.5	−1139.4	2639.2
BMI-for-age (percentile)					−31.0	33.4
Total energy (%EER)					10.6	29.9
Daily fat intake (%kcal)					356.9 *	156.3
*R* ^2^	0.01		0.16		0.19	
*R*^2^ change			0.15 ***		0.03	
Log 24H FV cups	8016.0 **	2612.3	7458.7 **	2510.9	9637.6 ***	2697.1
Age (years)			−1096.4 **	331.3	−1072.7 **	328.8
White (ref)						
Black/African-American			−1546.4	3890.4	−2028.0	3842.0
Multi-racial/other			51.1	2553.9	−820.0	2522.1
Hispanic			−4089.0	4546.2	−3405.3	4472.9
New York (ref)						
North Carolina			−11,104.0 **	3434.5	−12,005.4 ***	3399.4
Vermont			−2578.0	2379.7	−3113.0	2343.1
Washington			−6382.2 *	2525.3	−7049.3 **	2513.8
Smoker(s) in household			−824.3	2589.1	−933.0	2541.1
BMI-for-age (percentile)					−20.7	32.2
Total energy (%EER)					−27.0	30.8
Daily fat intake (%kcal)					486.8 **	155.2
*R* ^2^	0.05		0.20		0.25	
*R*^2^ change			0.15 ***		0.05 *	

NCI FV cups, National Cancer Institute’s All-Day Fruit and Vegetable Screener cup equivalents; BMI, body mass index; EER, estimated energy requirement; kcal, kilocalories; 24H FV cups, mean 24 h recall fruit and vegetable cup equivalents. * *p* < 0.05, ** *p* < 0.01, *** *p* < 0.001. Each model was run using multiple linear regression and controlled for each covariate with an estimate presented.

**Table 3:** Predicted RRS scores for discrete FV consumption levels among children under three scenarios (*n* = 177).

	Unadjusted Model		Field-Based Model		Research-Setting Model	
	Predicted Value	Margin of Error (±)	Predicted Value	Margin of Error (±)	Predicted Value	Margin of Error (±)
FV cups						
0.5	30,489.8	4667.1	30,902.2	4467.9	29,133.8	4728.9
1.0	32,795.9	3378.4	33,048.0	3221.6	31,906.4	3363.1
2.0	36,046.1	2066.4	36,072.2	1943.8	35,814.1	1924.8
3.0	38,352.2	2121.8	38,218.0	1998.9	38,586.7	1991.0
4.0	40,140.9	2777.0	39,882.3	2639.2	40,737.2	2719.9
5.0	41,602.4	3507.0	41,242.2	3347.9	42,494.4	3507.2

FV cups presented were modeled from 24 h recalls. The unadjusted model included no covariates. The field-based model included adjustments for age, race, ethnicity, state, and a smoker in the household. The research-setting model included all covariates in the field-based model and BMI-for-age percentile, energy intake, and fat intake.

## Data Availability

The data presented in this study are available on request from the corresponding author.
